# The lysosomal protein ABCD4 can transport vitamin B_12_ across liposomal membranes *in vitro*

**DOI:** 10.1016/j.jbc.2021.100654

**Published:** 2021-05-03

**Authors:** Katsuki Kitai, Kosuke Kawaguchi, Takenori Tomohiro, Masashi Morita, Takanori So, Tsuneo Imanaka

**Affiliations:** 1 Graduate School of Medicine and Pharmaceutical Sciences, University of Toyama, Toyama, Japan; 2 Faculty of Pharmaceutical Sciences, Hiroshima International University, Kure, Japan

**Keywords:** ABC transporter, lysosome, cobalamin, recombinant protein expression, protein purification, methylotrophic yeast, proteoliposome, β-DDM, *n*-dodecyl-β-d-maltoside, ER, endoplasmic reticulum, GST, glutathione-*S*-transferase, HA, hemagglutinin, NBD, nucleotide-binding domain, SDA, succinimidyl 4,4'-azipentanoate, TM3, transmembrane helix 3, TM6, transmembrane helix 6

## Abstract

Vitamin B_12_ (cobalamin) is an essential micronutrient for human health, and mutation and dysregulation of cobalamin metabolism are associated with serious diseases, such as methylmalonic aciduria and homocystinuria. Mutations in *ABCD4* or *LMBRD1*, which encode the ABC transporter ABCD4 and lysosomal membrane protein LMBD1, respectively, lead to errors in cobalamin metabolism, with the phenotype of a failure to release cobalamin from lysosomes. However, the mechanism of transport of cobalamin across the lysosomal membrane remains unknown. We previously demonstrated that LMBD1 is required for the translocation of ABCD4 from the endoplasmic reticulum to lysosomes. This suggests that ABCD4 performs an important function in lysosomal membrane cobalamin transport. In this study, we expressed human ABCD4 and LMBD1 in methylotrophic yeast and purified them. We prepared ABCD4 and/or LMBD1 containing liposomes loaded with cobalamin and then quantified the release of cobalamin from the liposomes by reverse-phase HPLC. We observed that ABCD4 was able to transport cobalamin from the inside to the outside of liposomes dependent on its ATPase activity and that LMBD1 exhibited no cobalamin transport activity. These results suggest that ABCD4 may be capable of transporting cobalamin from the lysosomal lumen to the cytosol. Furthermore, we examined a series of *ABCD4* missense mutations to understand how these alterations impair cobalamin transport. Our findings give insight into the molecular mechanism of cobalamin transport by which ABCD4 involves and its importance in cobalamin deficiency.

The ABC transporters comprise a superfamily of membrane-bound proteins that exist in almost all organisms from eubacteria to mammals. In humans, there are 48 members classified into the seven subfamilies A to G, based on structural organization and amino acid homology. These proteins catalyze the ATP-dependent transmembrane transport of a wide variety of substrates in order to sustain cellular homeostasis. Defect in their functions results in various inherited metabolic diseases (1, 2).

To date, four ABCD proteins, ABCD1‒4, have been identified in humans (3, 4, 5, 6). They are half-sized ABC proteins and mainly exist in the form of a homodimer (7). ABCD1‒3 are known to be peroxisomal proteins and involved in the transport of CoA derivatives of long, very long, or branched-chain fatty acids (8, 9). On the other hand, ABCD4 is located on lysosomal membrane (10). ABCD4 only slightly interacts with peroxisomal biogenesis factor Pex19p because of lack of the NH_2_-terminal hydrophobic region responsible for peroxisomal targeting. As a result, ABCD4 is recognized by signal recognition particles and integrated into the endoplasmic reticulum (ER) membrane (11). The ABCD4 dimer then forms a complex with lysosomal membrane protein LMBD1, and this complex is translocated from the ER to lysosomes (12).

ABCD4 is reportedly involved in the transport of vitamin B_12_ (cobalamin) from the lysosomal lumen to the cytosol, since the dysfunction of ABCD4 results in the failure of lysosomal cobalamin efflux (10). In humans, cobalamin binds with transcobalamin in the blood stream, and this complex is taken up into lysosomes by transcobalamin receptor–mediated endocytosis. Subsequently, cobalamin is released into the cytosol and converted into two active cofactors: methylcobalamin and adenosylcobalamin, which are required by the cytosolic enzyme methionine synthase and the mitochondrial enzyme methylmalonyl-CoA mutase, respectively. These cofactors are indispensable for the homeostasis of homocysteine and methylmalonic acid (13). As mentioned previously, mutations of *ABCD4* result in the failure of the release of cobalamin from lysosomes into the cytosol. A similar phenotype is caused by mutations of *LMBRD1*, which encodes the lysosomal membrane protein LMBD1 (14). LMBD1 shares a high degree of homology with the limb region protein (15) and lipocalin-1 interacting membrane receptor (16). It is reported that a small portion of LMBD1 exists on plasma membranes and is involved in the clathrin-mediated endocytosis of the insulin receptor as a specific adaptor (17). Since mutations of ABCD4 and LMBD1 result in a quite similar phenotype, these two proteins are thought to function as a complex during cobalamin metabolism.

We previously demonstrated that ABCD4 and LMBD1 form a complex on the ER membrane, and then this complex is translocated from the ER to lysosomes. In addition, this targeting ability of LMBD1 is indispensable for the targeting of ABCD4 to lysosomes (12). Thus, LMBD1 seems necessary to translocate ABCD4 to lysosomes from the ER, and ABCD4 plays a key role in the transport of cobalamin from the lysosomal lumen to the cytosol. Almost all mammalian ABC transporters, with some notable exception, localized on plasma and organelle membranes, transport the substrate from the cytosolic side to the extracellular space or the organelle lumen, which is considered to be the outside domain of cells; that is, they function as exporters (18). If ABCD4 actually transports cobalamin in this direction, ABCD4 would be the first example of a mammalian ABC importer that transports a soluble compound from the outside of cells.

In this study, to clarify the role of ABCD4 and LMBD1 in the transport of cobalamin, we expressed human ABCD4 and LMBD1 using the methylotrophic yeast *Komagataella phaffii* (formerly called *Pichia pastoris*) and reconstituted purified ABCD4 and/or LMBD1 in liposomes, as reported previously (19). Furthermore, we established a cobalamin transport assay employing ABCD4- and/or LMBD1-containing liposomes and characterized the transport mechanism. These results show that ABCD4 by itself transports cobalamin from the inside to the outside of liposomes and that LMBD1 is not required for the transport of cobalamin. These results indicate that ABCD4 functions as an importer on lysosomal membranes. Moreover, insights into the molecular mechanisms of the effect of mutant ABCD4 reported in cobalamin deficiency are provided.

## Results

### Reconstitution of purified ABCD4 in liposomes

**Figure 1:**
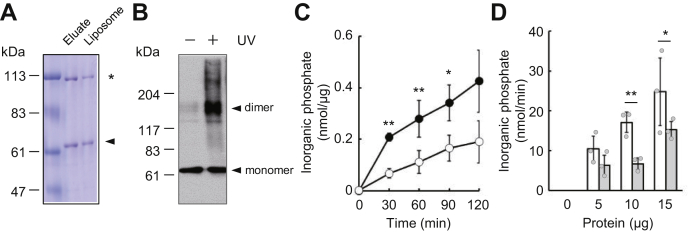
**ATPase activity of the reconstituted ABCD4.***A*, purified His-ABCD4 (Eluate) and reconstituted His-ABCD4 (liposomes) were subjected to SDS-PAGE, and the gel was stained with Coomassie brilliant blue. The *arrow head* and *asterisk* indicate His-ABCD4 and a nonspecific protein, respectively. *B*, photocrosslinking of His-ABCD4 was performed with succinimidyl 4,4'-azipentanoate (SDA) and UV irradiation. His-ABCD4 was detected by immunoblot analysis with an anti-His antibody. *C*, ATPase activity of reconstituted ABCD4 was measured. Proteoliposomes containing ABCD4 (6.96 μg) or negative control liposomes containing nonspecific protein (5.39 μg) were incubated with 5 mM ATP at 37 °C, and the phosphate that was released was measured. The ATPase activity of ABCD4 (•) and the negative control (○) are shown. Error bars indicate the standard deviation (n = 3). Differences between the ABCD4 and negative control were considered significant when *p* < 0.05 or *p* < 0.01 based on Student's *t* test (∗*p* < 0.05; ∗∗*p* < 0.01). *D*, different amounts of ABCD4-liposomes or negative control liposomes were incubated with 5 mM ATP at 37 °C for 30 min, and the phosphate that was released was measured. The ATPase activities of ABCD4 (*open bar*) and a nonspecific protein (*gray bar*) are shown. Error bars indicate the standard deviation (n = 3). Differences between ABCD4 and the negative control were considered significant when *p* < 0.05 or *p* < 0.01 based on Student's *t* test (∗*p* < 0.05; ∗∗*p* < 0.01).

ABCD4 and LMBD1 form a complex on lysosomal membranes and are involved in the transport of cobalamin transport across the lysosomal membrane (10, 14). ABCD4 is deduced to be involved in the transport of cobalamin, since LMBD1 assists translocation of ABCD4 from the ER to lysosomes (12). To test whether ABCD4 by itself is sufficient to transport cobalamin, we purified His-tagged human ABCD4 expressed in *K. phaffii* and reconstituted it in liposomes according to the procedure we previously established (19). His-ABCD4 expressed under the control of the strong methanol-inducible *AOX1* promoter in *K. phaffii* was solubilized by *n*-dodecyl-β-d-maltoside (β-DDM) and then purified by affinity chromatography (Fig. S1*A*). Since ABCD4 functions as a homodimer in mammalian cells (9, 20), we examined whether ABCD4 heterologously expressed in the yeast forms dimer or not by size exclusion chromatography. As shown in Fig. S1*B*, ABCD4 showed a polydisperse profile and ∼360 kDa major peak. The molecular weight of dimeric ABCD4 deduced from amino acid sequence is around 140 kDa, but ∼360 kDa major peak corresponds to dimeric ABCD4 as reported previously (20). Subsequently, purified His-ABCD4 was reconstituted in liposomes (Fig. 1*A*).

To examine proper insertion of ABCD4 on liposomes, we evaluated the proportion of the right-side-out orientation. ABCD4-liposomes were treated with trypsin and subjected to immunoblot analysis using anti-ABCD4 antibody recognizing C-terminal 149 amino acid of ABCD4. As shown in Fig. S2*A*, approximately 40 kDa band emerged. This band seems to be derived from ABCD4 existing in inside-out orientation. Approximately 70% of ABCD4 reconstituted into liposomes existed in right-side-out orientation calculated from the signal intensities (Fig. S2*B*). The 110 kDa band is a nonspecific protein derived from the yeast cells during His-tag affinity chromatography. This protein is not an ABCD4-associated protein because it was not coimmunoprecipitated with ABCD4 (Fig. S3*A*). Since this protein is also reconstituted in liposomes, the liposomes containing only this protein were used as a negative control (Fig. S3*B*).

As mentioned previously, ABCD4 functions as a homodimer in mammalian cells (9, 20). We examined whether ABCD4 is reconstituted in liposomes as a dimer or not. ABCD4-liposomes were incubated with succinimidyl 4,4'-azipentanoate (SDA), a cross-linking reagent. As shown in Figure 1*B*, ABCD4 displayed a ∼140 kDa band consistent in mass with dimeric ABCD4 when SDA was photoactivated by UV irradiation. Subsequently, the ATPase activity of liposome-reconstituted ABCD4 was assayed based on the release of organic phosphate from ATP. The reconstituted ABCD4 exhibited ATPase activity, and the activity was increased in a time- and dose-dependent manner (Fig. 1, *C* and *D*). These results indicate that ABCD4 was successfully reconstituted in liposomes in the active form.

### ABCD4 transports cobalamin as substrate

**Figure 2:**
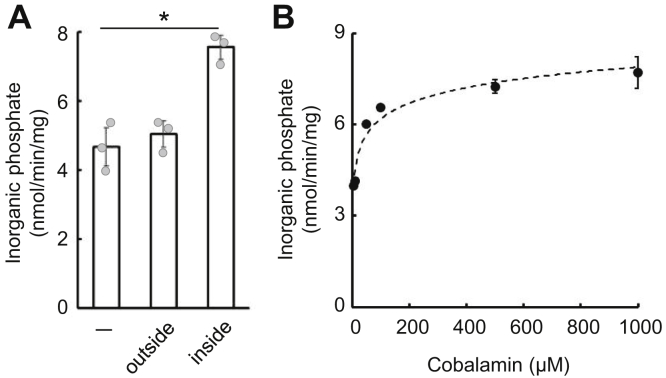
**Effect of cobalamin on the ATPase activity of ABCD4.***A*, ATPase activity of reconstituted ABCD4 was measured in the presence of 1 mM cobalamin inside or outside of the liposomes. Proteoliposomes with or without cobalamin containing ABCD4 (6.76 μg or 5.08 μg, respectively) were incubated with ATP at 37 °C for 30 min. Error bars indicate the standard deviation (n = 3). Differences among the conditions were considered significant when *p* < 0.05 based on Student's *t* test (∗*p* < 0.05). *B*, ATPase activity of reconstituted ABCD4 was measured in the presence of various concentrations of cobalamin in the liposome lumen. Error bars indicate the standard deviation (n = 3), when not shown, fall within the symbol.

As is well known, the ATPase activity of most ABC transporters is stimulated in the presence of transport substrate. We examined whether cobalamin stimulates the ATPase activity of ABCD4. As ABCD4 exits liposomes in both an inside-out and right-side-out orientation, we initially prepared ABCD4-liposomes with cobalamin and measured ATPase activities by the addition of ATP from outside the liposomes. The ATPase activity of ABCD4 was stimulated when cobalamin was present inside of the ABCD4-liposomes (Fig. 2*A*). On the other hand, we prepared ABCD4-liposomes without cobalamin and then measured the ATPase activity with cobalamin and ATP in the reaction mixture. As shown in Figure 2*A*, the addition of cobalamin did not stimulate the ATPase activity of ABCD4. Subsequently, we evaluated the ATPase activities of ABCD4-liposomes containing various concentrations of cobalamin. The stimulation of ABCD4 ATPase activity was increased in proportion to the concentration of cobalamin inside of the ABCD4-liposomes (Fig. 2*B*). These results indicate that the cobalamin inside liposomes is recognized by ABCD4 as substrate and stimulates the ATP hydrolysis of ABCD4.

**Figure 3:**
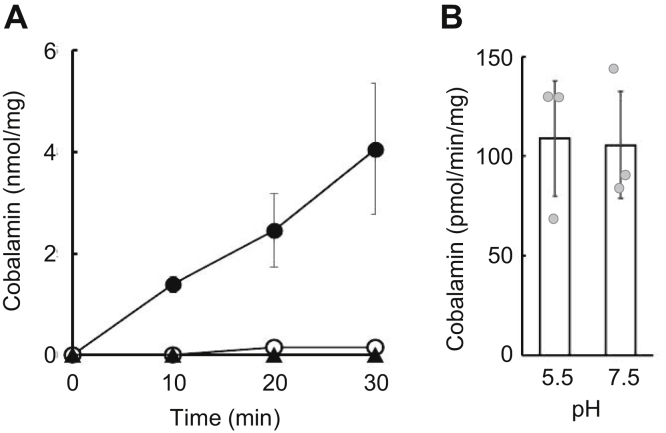
**Cobalamin transport activity of ABCD4.***A*, ABCD4-liposomes containing 100 μM of cobalamin were prepared. Proteoliposomes containing ABCD4 wildtype or K427A (5.54 μg or 4.95 μg, respectively) were incubated with ATP at 37 °C. The amount of cobalamin transported from inside of the liposomes at each time point was calculated by subtraction of the amount of cobalamin in the liposomes in each incubation from that without any incubation. The cobalamin transport activity of wildtype ABCD4 (•), ABCD4 (K427A) (▲), and the negative control (○) are shown. Error bars indicate the standard deviation (n = 3), when not shown, fall within the symbol. *B*, cobalamin containing ABCD4-liposomes with different internal pH conditions was incubated with ATP, and then the amount of cobalamin inside of the liposomes was quantified as in (*A*). Proteoliposomes at pH 5.5 or pH 7.5 containing ABCD4 (10.1 μg or 10.2 μg, respectively) were used for this assay. Error bars indicate the standard deviation (n = 3), when not shown, fall within the symbol.

Next, we performed a cobalamin transport assay using ABCD4-liposomes loaded with cobalamin. After incubation with ATP for 30 min, cobalamin was released from ABCD4-liposomes but not negative control liposomes (Fig. S4). Subsequently, the ABCD4-liposomes were incubated with ATP, and aliquots were removed at various time points. The cobalamin released from the ABCD4-liposomes was calculated as described in Experimental procedures section. As shown in Figure 3*A*, cobalamin was transported in a time-dependent manner by ABCD4. Kinetic properties were determined using ABCD4 containing liposomes loaded with various concentrations of cobalamin. Lineweaver–Burk plot was used to calculate the *K*_*m*_ and *V*_max_ values, which came out as 426 μM and 667 pmol/min/mg, respectively (Fig. S5). Since the lysosome lumen is maintained in an acidic condition, we next examined the effect of a low pH inside the liposomes on the transport activities of ABCD4. ABCD4-liposomes were prepared in an acidic pH, and the transport assay was performed at a neural pH to mimic the physiological conditions. However, the low pH condition did not exert any influence on either the ATPase or the cobalamin transport activities (Fig. 3*B* and Fig. S6), suggesting that cobalamin transport by ABCD4 is not dependent on proton-motive force.

Next, to test whether cobalamin transport depends on ATPase activity, we prepared ABCD4 (K427A), a Walker A lysine mutant deficient in ATPase activity. We also expressed His-ABCD4 (K427A) under the control of the *AOX1* promoter in *K. phaffii*. His-ABCD4 (K427A) was purified and reconstituted in liposomes using the same procedure as used for His-ABCD4 (Fig. S7*A*). It was confirmed that this mutant had lost ATPase activity (Fig. S7*B*). In the transport assay, cobalamin was not released from ABCD4 (K427A)-liposomes loaded with cobalamin at any time point (Fig. 3*A*). This indicates that ABCD4 (K427A) had lost transport activity in addition to ATPase activity. These results demonstrated that ABCD4 transports cobalamin from the inside to the outside of liposomes in a manner that is dependent on ATPase activity.

### Effect of LMBD1 on the function of ABCD4

**Figure 4:**
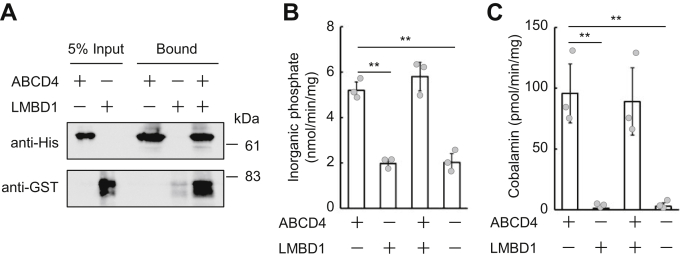
**Effect of LMBD1 on the enzyme activities of ABCD4.***A*, interaction analysis between purified ABCD4 and LMBD1. The eluates of His-ABCD4 and LMBD1–glutathione-*S*-transferase (GST) were mixed and rotated at 4 °C for 15 min. Then the mixture was incubated with His-tag affinity resin overnight at 4 °C. Coprecipitated proteins were analyzed by SDS-PAGE followed by immunoblotting using an anti-His or an anti-GST antibody. The ATPase activity (*B*) and cobalamin transport activity (*C*) of ABCD4 and/or LMBD1 liposomes were evaluated. Proteoliposomes containing ABCD4 (3.91 μg), LMBD1 (3.75 μg), or ABCD4 and LMBD1 (2.34 and 2.54 μg, respectively). Error bars indicate the standard deviation (n = 3). Differences among the liposomes were considered significant when *p* < 0.01 based on Student's *t* test (∗∗*p* < 0.01).

As described previously, mutations in *LMBRD1*, which encodes the lysosomal membrane protein LMBD1, result in the failure of cobalamin release from lysosomes, which is the same phenotype as seen in ABCD4 dysfunction. We previously demonstrated that LMBD1 interacts with ABCD4 and regulates the translocation of ABCD4 from the ER to lysosomes in mammalian cells (12), suggesting that mutation of LMBD1 affects this translocation of ABCD4. We examined whether LMBD1 has any other effect on ABCD4 function in addition to its localization. We first prepared a *K. phaffii* strain expressing glutathione-*S*-transferase (GST)–tagged LMBD1. The expressed LMBD1–GST was solubilized with β-DDM and then purified by GST-tag affinity chromatography (Fig. S8). Then we examined whether purified ABCD4 and LMBD1 interact with each other. His-ABCD4 and LMBD1–GST were mixed and then subjected to pull-down assay using His-tag affinity resin. As shown in Figure 4*A*, LMBD1–GST was coprecipitated with His-ABCD4. Likewise, His-ABCD4 was coprecipitated with LMBD1–GST by pull-down assay using GST-tag affinity agarose (Fig. S9*A*). In addition, LMBD1 was not coprecipitated with His-ABCD1 unlike ABCD4 (Fig. S9*B*), suggesting that interaction of ABCD4 with LMBD1 is specific, and it is not nonspecific interactions between two misfolded membrane proteins. Furthermore, the binding stoichiometry between purified ABCD4 and LMBD1 was analyzed by crosslinking. As shown in Fig. S9*C*, ∼300 kDa band consistent in mass with one dimeric His-ABCD4 plus one dimeric LMBD1–GST was detected in addition to dimeric His-ABCD4 or dimeric LMBD1–GST when cross-linking reagents were photoactivated by UV irradiation. These results indicate that purified ABCD4 and LMBD1 interact each other with a 1:1 stoichiometry.

Subsequently, we prepared ABCD4/LMBD1-liposomes. It was confirmed that ABCD4 and LMBD1 maintained the same binding stoichiometry in ABCD4/LMBD1-liposomes (Fig. S9*C*). ABCD4/LMBD1-liposomes also exhibited both ATPase and cobalamin transport activity. There was no difference in either the ATPase or the cobalamin transport activities compared with the ABCD4-liposomes (Fig. 4, *B* and *C*). LMBD1 itself had neither ATPase nor cobalamin transport activities (Fig. 4, *B* and *C*).

### Dysfunction of ABCD4 bearing disease-related missense mutations

To date, eight clinical mutations in the *ABCD4* gene that result in cobalamin deficiency have been reported (21). Among them, there are three missense mutations, N141K, Y319C, and R432Q. Arg^432^ is located on or very close to the Walker A motif, which is indispensable for ATPase activity, and it is reported that the R432Q mutant exhibits much lower ATPase activity (22). It is also reported that Asn^141^ and Tyr^319^ are located on the cytosolic side of transmembrane helix 3 (TM3) and the lysosomal side of transmembrane helix 6 (TM6), respectively, as shown by cryo-EM (22). We focused on the role of these amino acid residues during the transport of cobalamin mediated by ABCD4.

We initially examined the subcellular localization of ABCD4 (N141K) and ABCD4 (Y319C) in mammalian cells. We previously established CHO cells stably expressing LMBD1–GFP and demonstrated that the ABCD4–hemagglutinin (HA) transiently expressed in these cells is localized to lysosomes (12). ABCD4 (N141K)–HA or ABCD4 (Y319C)–HA was also transiently expressed in these CHO cells. The distribution of ABCD4 (N141K)–HA or ABCD4 (Y319C)–HA partially coincided with that of LMBD1–GFP (Fig. S10), indicating that a portion of both ABCD4 (N141K) and ABCD4 (Y319C) is localized in lysosomes, as in the case of wildtype ABCD4.

**Figure 5:**
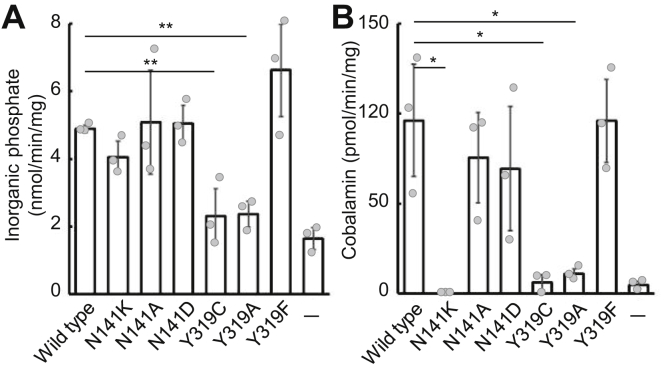
**ATPase and cobalamin transport activities of mutant ABCD4.** The ATPase activity (*A*) and cobalamin transport activity (*B*) of mutant ABCD4 were evaluated. Error bars indicate the standard deviation (n = 3). Proteoliposomes containing ABCD4 wildtype, N141A, N141D, N141K, Y319A, Y319C, or Y319F (6.62, 6.79, 7.63, 6.49, 5.35, 6.38, or 9.48 μg, respectively) were used for these assays. Differences between the wildtype and mutant were considered significant when *p* < 0.05 or *p* < 0.01 based on Student's *t* test (∗*p* < 0.05; ∗∗*p* < 0.01).

Next, to evaluate the function of ABCD4 (N141K) and ABCD4 (Y319C), we prepared a *K. phaffii* strain expressing His-ABCD4 (N141K) or His-ABCD4 (Y319C). Both types of mutant ABCD4 were purified and reconstituted in liposomes using the same procedure as used for His-ABCD4 (Fig. S11*A*). Moreover, it was confirmed that these mutants were reconstituted in liposomes as a dimer (Fig. S11*B*). Subsequently, the ATPase and transport activities of these mutants were examined. As shown in Figure 5, *A* and *B*, ABCD4 (N141K) exhibited ATPase activity corresponding with the wildtype but lost cobalamin transport activity. On the other hand, ABCD4 (Y319C) lost both the ATPase and transport activities.

Asn^141^ is located on the cytosolic side of TM3 and faces the transmembrane cavity and is deduced to form a hydrogen bond with Asp^221^, which is located on the cytosolic side of TM4 (22). The hydrophobic or ionic environment of this area might be important for the transport of cobalamin through the conformational change of the TMs associated with the ATP binding and hydrolysis at the nucleotide-binding domain (NBD). We changed the mutated Lys^141^ to Ala^141^ or Asp^141^ and measured the ATPase and cobalamin transport activities. As shown in Figure 5*A*, ABCD4 (N141A) and ABCD4 (N141D) also exhibited ATPase activity equal to that of the wildtype. Interestingly, the cobalamin transport activity was recovered to the same level as the wildtype in both ABCD4 (N141A) and ABCD4 (N141D) (Fig. 5*B*). These results indicate that an increase in the anionic charge in this area by substitution of Asn^141^ to Lys^141^ might disturb the cobalamin transport of ABCD4.

In the case of Tyr^319^, substitution to Cys resulted in the loss of the ATPase activity as well as cobalamin transport activity. Surprisingly, Tyr^319^ is located on the lumen side of TM6 far away from the NBD that is indispensable for ATPase activity (22). To evaluate the role of Tyr^319^, we first tested whether ATP can access the NBD in ABCD4 (Y319C) using an ATP probe for photoaffinity labeling (Fig. S12*A*), since ABCD4 (Y319C) lacks ATPase activity. As a result, ABCD4 (Y319C) was labeled by the ATP probe under UV irradiation, and this labeling was inhibited by the presence of adenylyl imidodiphosphate, a nonhydrolyzable analog of ATP, in a dose-dependent manner (Fig. S12*B*). These results suggest that the conformational change coupled to the hydrolysis of ATP was disturbed in the Y319C mutant. Recently, in *Cyanidioschyzon merolae* ABCB1, the van der Waals contacts and hydrogen-bonding network in the cluster of aromatic hydrophobic side chains in the upper part of the transmembrane helices were shown to play an important role in the interactions among the transmembrane helices (23, 24). The Tyr^319^ residue in ABCD4 is deduced to be involved in the corresponding cluster. Therefore, we prepared ABCD4 (Y319A) and ABCD4 (Y319F) and evaluated both the ATPase and cobalamin transport activities. As shown in Figure 5, *A* and *B*, neither the ATPase activity nor the transport activity was recovered in ABCD4 (Y319A). In contrast, ABCD4 (Y319F) recovered both the ATPase and transport activities (Fig. 5, *A* and *B*). These results indicate that Tyr^319^ in ABCD4 plays an important role in the conformational change related to cobalamin transport.

## Discussion

Since mutations in the genes *ABCD4* and *LMBRD1*, which encode ABCD4 and LMBD1, respectively, result in a failure to release cobalamin from lysosomes (10, 14), ABCD4 and LMBD1 are considered to be coordinatively involved in the transport of cobalamin across the lysosomal membrane. We previously demonstrated that translocation of ABCD4 from the ER to lysosomes is assisted by LMBD1 since ABCD4 itself only targets the ER (12). However, ABCD4 exists in a complex with LMBD1 on the lysosomal membrane, and the molecular mechanism of cobalamin transport mediated by ABCD4/LMBD1 has yet to be elucidated including whether ABCD4 is by itself sufficient for cobalamin transport. Previously, we reconstituted purified ABCD4 in liposomes and showed ABCD4 possesses ATPase activity. Here, we characterized transport mechanism of cobalamin by ABCD4 with or without LMBD1 and how the transport is impaired by disease-related mutations of ABCD4.

In our assay system, the ATPase activity of ABCD4 was stimulated only when cobalamin is present inside the liposomes and ATP was added to the outside (Fig. 2). In addition, ABCD4 was able to transport cobalamin from the inside to the outside of liposomes in a manner that was dependent on its ATPase activity (Fig. 3*A*). It is predicted that the COOH-terminal half of ABCD4, including the NBD, is exposed to the cytosolic surface (10, 11). Therefore, this system reproduces the transport of cobalamin from the lysosomal lumen to the cytosol. The transport of one molecule of cobalamin requires the hydrolysis of approximately 50 molecules of ATP. This estimate is comparable to that of the transport of several different substances by the multidrug resistance protein 1/ABCC1 reconstituted in liposomes (25, 26). ABCC1 is known to export cobalamin into the extracellular space from the cytosol (27). Recently, as ABCD4 and LMBD1 have been shown to interact with the cobalamin processing proteins, methylmalonic aciduria and homocystinuria type C protein and methylmalonic aciduria and homocystinuria type cblD (20), it has been hypothesized that cobalamin might be transported more efficiently by ABCD4 from lysosomes *in vivo*.

Concerning the direction of the transport carried out by the ABCD4 family of transporters, ABCD1‒3 transport fatty acid CoAs into peroxisomes from the cytosol. ABCD4 transports cobalamin from the lysosome lumen to opposite side. ABCD1‒4 are half-sized transporters and exist as a homodimer on the membrane. They exhibit the same orientation as that of the NBD, which is exposed to the cytosolic surface (10, 11). It is interesting that ABCD1‒3 and ABCD4 recognize substrate on the cytosolic or lumenal face of lysosomes, respectively, and transport these substrates in different directions *via* conformational changes of the transmembrane domain coupled with ATP binding and hydrolysis of the NBD. ABC transporters are categorized into two types: importers and exporters. ABC importers, which transport substrates into the cytosol from the extracellular space, are restricted to prokaryotes, with only a few exceptions in plants and mammals. Recently, ABCA4, cystic fibrosis transmembrane conductance regulator (ABCC7), SUR1 (ABCC8), and SUR2 (ABCC9) are categorized as importers (18). ABCA4 functions as flippase that translocates *N*-retinylidene-phosphatidylethanolamine from the lumen to the cytoplasmic leaflet of the disc membrane in retinal photoreceptor cells (28). Cystic fibrosis transmembrane conductance regulator functions as an ATP-gated chloride channel (29), and SUR1 and SUR2 are regulatory elements of the hetero-octameric ATP-sensitive potassium (K_ATP_) channels built from four SUR subunits (30). Therefore, ABCD4 is the first mammalian ABC importer shown to transport a soluble compound.

LMBD1 regulates the lysosomal translocation of ABCD4 and forms a complex with ABCD4 on the lysosomal membrane (12, 21). However, LMBD1 itself did not exhibit any cobalamin transport activity and also did not assist either the ATPase or the cobalamin transport activity of ABCD4 (Fig. 4, *B* and *C*). These results indicate that LMBD1 is not directly involved in cobalamin transport on the lysosomal membrane, although the dysfunction of LMBD1 did result in a failure in lysosomal cobalamin release. Lysosomes contain more than 60 hydrolases to degrade extracellular materials as well as intracellular components. Many of the lysosomal membrane proteins are highly glycosylated within the lysosomal lumen in order to let them escape lysosomal proteolysis (31). LMBD1 possesses six putative glycosylation sites and is, in fact, glycosylated (14, 20). In contrast, ABCD4 possesses two potential glycosylation sites but is not glycosylated in mammalian cells (11). It is likely that LMBD1 contributes to the protection of ABCD4 from lysosomal proteolysis by forming a complex with ABCD4.

There are eight clinically relevant mutations in *ABCD4*, including two in-frame deletions, three flame shifts, and three point mutations, N141K, Y319C, and R432Q. Arg^432^ is located next to the Walker A motif, and ABCD4 (R432Q) exhibits much lower ATPase activity than the wildtype (22). Since it was demonstrated that ABCD4 transports cobalamin in a manner that is dependent on ATPase activity (Fig. 3*A*), the defect of cobalamin metabolism in the R432Q mutant is almost certainly because of the loss of ATPase activity.

On the other hand, Asn^141^ and Tyr^319^ are located on the cytosolic side of TM3 and the lysosomal side of TM6, respectively. ABCD4 (N141K) lacks only cobalamin transport activity, whereas ABCD4 (Y319C) lacks both ATPase and cobalamin transport activity (Fig. 5, *A* and *B*). From the amino acid coordinates of ABCD4 deposited in the Protein Data Bank (6JBJ), Asn^141^ faces the transmembrane cavity (22). The hydrophobic or ionic environment of the area might be important for the transport of cobalamin *via* the conformational change that is associated with the ATP binding and hydrolysis of the NBD. Since transport activity was recovered in ABCD4 (N141A) and ABCD4 (N141D) (Fig. 5*B*), an increase in the cationic charge around Asn^141^ by substitution to Lys^141^ might disturb the cobalamin transport of ABCD4. Asn^141^ is believed to form a hydrogen bond with Asp^221^ located on the cytosolic side of TM4. However, the substitution of Asn^141^ to Ala^141^, which does not form a hydrogen bond with Asp^221^, did not influence the cobalamin transport activity of ABCD4. It is thus suggested that the hydrogen bond between Asn^141^ and Asp^221^ is not important for cobalamin transport. From the cryo-EM structure, the distance between Asn^141^ and Asp^221^ is very close (2.5 Å) (Fig. S13). Since the Lys residue possesses a large side chain with a positive charge, the local environment around Lys^141^ might be altered and TM3 becomes strained. Therefore, dimerization of the NBD accompanied by ATP hydrolysis might be linked with the conformational change in the transmembrane domain required to transport cobalamin.

Tyr^319^ is located on the lysosomal side of TM6 and deduced to be involved in the hydrophobic entrance of the transmembrane cavity (22). Since the Y319C mutant lacks the ATPase as well as transport activity (Fig. 5, *A* and *B*), disturbance in the accessibility of cobalamin to the cavity does not seem to be the exclusive reason for the impaired transport of cobalamin. It is speculated that in the Y319C mutant, the two Cys^319^ residues have a tendency to form a disulfide bond in a dimeric conformation (22) and, as a result, there is a certain impairment in the conformational change that might impact transport. However, disulfide bond formation is not the reason, since ABCD4 (Y319A) also lost ATPase as well as cobalamin transport activity (Fig. 5, *A* and *B*). ABCD4 (Y319C) was able to bind but not hydrolyze ATP (Fig. 5*A* and Fig. S12*B*). These results indicate that the Y319C mutant has an additional conformational change impairment. Recently, the inward- and outward-facing X-ray crystal structure of *C. merolae* ABCB1 was reported, and the structural features during conformational realignment revealed based on high-resolution crystallographic data (24). The aromatic amino acid residues in the upper part of the transmembrane helices of TM4 and TM6 form van der Waals contacts and hydrogen-bonding networks as a hydrophobic cluster and are involved in the extracellular gating that occurs during the conformational changes that take place along with ATP binding. ABCD4 possesses a similar cluster as TM4 and TM6. The Tyr^319^ residue in TM6 is deduced to form the cluster and play an important role during the conformational change associated with ATP binding and hydrolysis because the substitution of Tyr^319^ to Phe with an aromatic ring restored the ATPase as well as cobalamin transport activities of ABCD4 (Fig. 5, *A* and *B*). Further crystallographic study of ABCD4 will be needed.

In this study, it was demonstrated for the first time that ABCD4 transports cobalamin from the lysosomal lumen to the cytosol as an “importer.” In contrast, other members of the ABC transporter subfamily D, ABCD1‒3, are involved in the transport of fatty acyl-CoAs from the cytosol to the peroxisomal lumen. Furthermore, it is recently reported that some bacterial ABC transporters, which possess the same transmembrane folding with mammalian ABC exporters, function as an importer (32, 33). Elucidation of the mechanism underlying the direction of substrate transport is a subject for future investigation.

## Experimental procedures

### Yeast strains and media

*K. phaffii* strain SMD1168 *his4* was used as the host strain to express the human ABCD4 and LMBD1. The strains were grown in 1% yeast extract, 2% peptone, and 2% glucose or buffered minimal (0.5% yeast extract and 0.5% methanol) medium.

### Purification of His-ABCD4 and LMBD1–GST

The expressed proteins were purified according to the procedure previously described (19) with some modifications. Yeast cells expressing His-ABCD4 were grown to midlog phase on 1% yeast extract, 2% peptone, and 2% glucose medium. Subsequently, the cells were transferred to buffered minimal medium and incubated for 18 h at 30 °C. The cells were resuspended with Tris buffer (50 mM Tris–HCl at pH 7.5, 300 mM NaCl, and 5 mM DTT) and then disrupted with 0.3-mm zirconia beads in a Multi-Beads Shocker (YASUI KIKAI Co, Ltd). All purification steps were conducted at 4 °C. Undisrupted cells, nuclei, and other cell debris were removed by centrifugation at 1500*g* for 10 min. The resulting supernatant (cell-free extract) was subjected to centrifugation at 14,000*g* for 30 min to obtain an organelle pellet. Membranes were solubilized by 0.5% β-DDM for 3 h on an end-over-end rotator. Insoluble material was removed by centrifugation at 100,000*g* for 30 min. The supernatant was incubated with cOmplete His-Tag Purification Resin (Roche) with 5 mM imidazole on an end-over-end rotator for 16 h. Subsequently, the resin was washed twice with Tris buffer containing 0.1% β-DDM and 50 mM imidazole, and His-ABCD4 was eluted with Tris buffer containing 0.1% β-DDM and 500 mM imidazole.

To purify LMBD1–GST, solubilization of LMBD1–GST was performed by the same procedure as used for His-ABCD4. The supernatant after β-DDM treatment was incubated with COSMOGEL GST-Accept agarose (Nacalai Tesque, Inc). The resin was washed with Tris buffer and eluted with Tris buffer containing 50 mM glutathione.

Protein concentration in eluate fraction was determined by the method of Bradford. As His-ABCD4 and the nonspecific protein existed in the eluate fraction, the amount of His-ABCD4 was calculated from the ratio of the intensity of His-ABCD4 and the nonspecific protein in acrylamide gel after SDS-PAGE and Coomassie brilliant blue staining.

### Preparation of proteoliposomes

Liposomes were prepared as previously described (34). Soybean l-α-phosphatidylcholine (10 mg/ml, Type II-S; Sigma) was suspended in the suspension buffer (20 mM Tris–HCl at pH 7.5). The mixture was sonicated until clear in a bath-type sonicator and then frozen and thawed five times. Liposomes were stored at −80 °C until use. Aliquot of 50 μg of protein in eluate fraction was mixed with 500 μg of liposomes in a 1:10 ratio (W/W) and then frozen and thawed twice. The mixture was diluted 30-fold with the reconstitution buffer (20 mM Tris–HCl at pH 7.5 and 0.5 mM DTT). Reconstituted proteoliposomes were pelleted *via* centrifugation at 150,000*g* for 1 h at 4 °C and then suspended in 200 μl of 20 mM Tris–HCl at pH 7.5. To prepare proteoliposomes containing both ABCD4 and LMBD1, protein solutions of His-ABCD4 and LMBD1–GST were incubated for 1 h at 4 °C before being mixed with the liposomes. To prepare proteoliposomes containing cobalamin, we added cobalamin to both the suspension and the reconstitution buffer. The amount of ABCD4 incorporated into liposomes was calculated by immunoblot analysis of His-ABCD4 using His-ABCD4 in eluate fraction as standard. The signal strength of His-ABCD4 was quantified by the ImageJ image analysis software (NIH).

### ATPase activity

The ATPase activity of the reconstituted proteins in the liposomes was determined by measuring the release of inorganic phosphate from ATP, as previously described (35). Proteoliposomes were mixed with reaction buffer (50 mM Tris–HCl at pH 7.6, 300 mM NaCl, 11 mM MgCl_2_, 1.1 mM EGTA, 2.2 mM DTT, 10 mM sodium azide, and 2 mM ouabain). The reaction was started by the addition of ATP (final concentration of 5 mM) and incubated at 37 °C. This reaction was stopped by the addition of 1 N HCl and supplemented with malachite green solution (0.034% malachite green, 1.05% ammonium molybdate, and 0.1% TritonX-100). Absorption at 620 nm was measured after incubation at 30 °C for 30 min.

### Photocrosslinking

ABCD4-liposomes were incubated with SDA (Thermo Fisher Scientific) (final concentration of 2 mM) at room temperature for 30 min. The reaction was stopped by the addition of Tris–HCl at pH 8.8 at a final concentration of 200 mM and then incubated on ice for 15 min. SDA-labeled proteins were photoactivated by UV irradiation at 365 nm for 1 min. Samples were subjected to immunoblot analysis. To evaluate binding stoichiometry between ABCD4 and LMBD1, *m*-maleimidobenzoyl-*N*-hydroxysuccinimide ester (Thermo Fisher Scientific) (final concentration of 2 mM) was used in addition to SDA.

### Cobalamin transport assay

Proteoliposomes containing 100 μM of cobalamin were incubated with the reaction buffer (50 mM Tris–HCl at pH 7.6, 100 mM KCl, 20 mM MgCl_2_, 4 mM DTT, and 2 mM ATP) at 37 °C. Aliquots were removed at various time points and applied to a Sephadex G-50 column (GE Healthcare) to collect the proteoliposomes. The proteoliposomes were then disrupted with 1% Triton X-100 and applied on a COSMOSIL 5C_18_-MS-II column (4.6 × 25 cm; Nacalai Tesque, Inc) at a flow rate of 1 ml/min equilibrated with 20 mM ammonium acetate buffer. Elution was performed with a linear gradient of an increasing acetonitrile concentration (0‒5 min, 5%; 2‒25 min, 5‒40%; and 25‒30 min, 40%) in 20 mM ammonium acetate. Cobalamin was detected by UV 361 nm. The amount of cobalamin transported from inside of the liposomes at each time point was calculated by subtraction of the amount of cobalamin in the liposomes at each incubation from that without any incubation.

### Pull-down assay

The eluates of each protein (15 μg each) were mixed and rotated at 4 °C for 15 min. The mixture was then incubated with cOmplete His-tagged Resin or COSMOGEL GST-Accept agarose overnight at 4 °C. The resin or agarose was collected by centrifugation and washed twice with wash buffer (50 mM Tris–HCl at pH 7.5, 300 mM NaCl, and 0.1% β-DDM). The bound proteins were eluted in SDS contained in the sample buffer and subjected to immunoblot analysis.

## Data availability

All data are given in the main article or supporting information.

## Supporting information

This article contains supporting information (11, 12, 19, 36).

## Conflict of interest

The authors declare that they have no conflicts of interest with the contents of this article.
