# Supplementary material for: The lysosomal protein ABCD4 can transport vitamin B12 across liposomal membranes in vitro
Source: J Biol Chem. 2021 May 3;296:100654. doi: 10.1016/j.jbc.2021.100654 (PMC8113721; doi:10.1016/j.jbc.2021.100654)
Supplement: Supplemental Figures S1–S13 [file mmc1.pdf]

The lysosomal protein ABCD4 can transport vitamin B_{12} across liposomal membranes *in vitro*

Katsuki Kitai[1,**], Kosuke Kawaguchi[1,*,**], Takenori Tomohiro [1], Masashi Morita[1], Takanori So[1] and Tsuneo Imanaka[2]

[1]Graduate School of Medicine and Pharmaceutical Sciences, University of Toyama, Toyama, Japan.
[2]Faculty of Pharmaceutical Sciences, Hiroshima International University, Kure, Japan

[*] Corresponding author: Kosuke Kawaguchi
E-mail: kkawa@pha.u-toyama.ac.jp

[**]These authors contributed equally to this work.

Running title: ABCD4 transports vitamin B_{12}

Keywords: ABC transporter; lysosome; cobalamin; recombinant protein expression; protein purification; methylotrophic yeast; proteoliposome

Experimental procedures

Cell culture conditions and the transfection procedure

Chinese hamster ovary (CHO) cells were cultured in Dulbecco's modified Eagle medium (DMEM) supplemented with 10% fetal bovine serum, 100 units/ml penicillin and 140 μg/ml streptomycin sulfate at 37°C and 5% CO_2. All of the transfection procedures were performed using the Effectene Transfection Reagent (Qiagen, Hilden, Germany) according to the manufacturer's instructions.

Plasmid construction

The oligonucleotide primers used for the PCR reactions are listed in Table S1. The plasmid for expressing human ABCD4, pIB4-His-ABCD4, was constructed previously (1). The plasmid for expressing ABCD4(N141K) was constructed as follows. A 7.7-kb fragment was amplified by inverse PCR with the primer sets Fw-ABCD4-N141K/Rv-ABCD4-N141 using pIB4-His-ABCD4 as the template, and then this fragment was self-ligated using T4 Polynucleotide Kinase (Toyobo, Osaka, Japan) and Ligation high (Toyobo) to form pIB4-His-ABCD1(N141K). All of the mutant ABCD4-expressing plasmids were constructed following the procedure described above.

The human *LMBRD1* gene, which encodes LMBD1, was synthesized according to the preferred codon usage of *K. phaffii* by GenScript. The sequence was submitted to DDBJ under accession no. LC516919. The synthesized LMBD1 fragment was digested with *Kpn*I and *Xho*I and ligated with the *Kpn*I/*Xho*I fragment of pIB4 to yield pIB4-sLMBD1. Next, a glutathione *S*-transferase (GST) tagged LMBD1 expression vector was constructed. The 7.5-kb fragment was amplified by inverse PCR using the primer set Fw-inv-LMBD1/Rv-inv-LMBD1 and pIB4-sLMBD1 as the template. The 0.73-kb fragment coding GST was amplified using the primer set Fw-infu-GST/Rv-infu-GST and pGEX6P-1 as the template. These two fragments were fused using an In-Fusion HD cloning kit (TaKaRa, Shiga, Japan) to yield pIB4-LMBD1-GST.

The HA-tagged ABCD4 expressing vector pcDNA3.1-ABCD4-HA was constructed previously (2). The mutant ABCD4 expressing plasmids were constructed following the same

procedure for yeast cells described avobe, using pcDNA3.1-ABCD4-HA as the template.

Gel filtration chromatography

Gel filtration chromatography was performed by essentially the same procedure as described previously (3). The eluate of His-ABCD4 (200 μg) from the His-Tag Purification Resin was applied to a Superdex 200 10/300 GL column (GE Healthcare) equilibrated with gel filtration buffer (20 mM Tris-HCl pH 7.5, 150 mM NaCl, 5 mM DTT and 0.1% β-DDM). Gel filtration analysis was performed at room tempreture with a flow rate of 0.3 ml/min and fractions (0.5 ml) were corrected. The collected fractions were subjected to SDS-PAGE followed by immunoblot analysis.

Co-Immunoprecipitation

The eluate fraction (100 μg) containing His-ABCD4 and non-specific protein was incubated with 5 μg of anti-His antibody (FUJIFILM Wako, Osaka, Japan) or Ig for overnight, and then incubated with Protein G-Agarose beads (Sigma Aldrich, St. Louis, MO) for 1 h. The Protein G-Agarose beads were collected by centrifugation and washed three times with the buffer (20 mM Tris-HCl pH 7.5, 300 mM NaCl, 5 mM DTT and 0.1% β-DDM). The immunoprecipitated proteins were subjected to SDS-PAGE followed by CBB staining.

Orientation of ABCD4 reconstituted into liposomes

ABCD4-liposomes were incubated with or without 50 ng of trypsin, or with trypsin in the presence of 0.05% Triton X-100 for 30 min on ice. Samples were immediately subjected to SDS-PAGE followed by immunoblot analysis using anti-ABCD4 antibody recognizing C-terminal 149 amino acid (4). The intensity of the bands corresponding ABCD4 and its fragments was quantified by the image analysis software Image J.

Indirect immunofluorescence

Immunostaining was performed by essentially the same procedure as described previously (2). The fixed cells were permeabilized in PBS containing 1% (w/v) Triton X-100 for 5 min or 30 μM digitonin for 15 min, washed three times with PBS, and incubated with the primary antibodies for 45 min at room temperature. The primary antibodies in this study were

used in combination with a mouse antibody against HA and a rabbit antibody against LAMP1. Cy3-conjugated goat anti-mouse IgG and Cy5-conjugated goat anti-rabbit IgG were used to mark the primary antibodies. The cells were mounted in Slowfade Gold Antifade Reagent (Thermo Fisher Scientific, Waltham, MA) and the samples were observed under a Zeiss LSM780 laser scanning confocal microscope equipped with a Plan-APOCHROMAT 20x, NA 0.8 objective on an inverted microscope Axio Observer Z1(Carl Zeiss, Oberkochen, Germany). Cy3 and Cy5 were excited with 543 nm and 633 nm HeNe lasers, respectively.

Construction of the ATP probe

The scheme used for constructing the ATP probe is shown in Fig. S12A.

To synthesize Compound **2**, ATP (55.1 mg, 100 µmol) and 1-ethyl-3-(3-dimethylaminopropyl)carbodiimide hydrochloride (EDCI, 86.2 mg, 450 µmol) were incubated in a 1 ml solution (0.1 M $NaHCO_3$, pH 7.5) for 5 min at room temperature. A DMF solution (1.8 ml) of compound **1** (13.7 mg, 100 µmol) was added and stirred at room temperature overnight. 100 µl of 1 M NaOH was then added to the solution and stirred for 1 hour. The product was purified by reverse-phase HPLC on ODS (SHISEIDO CAPCELLPAK C18, 5 µm, 10 mm x 250 mm) with a liner gradient of 0–40% acetonitrile-water containing 50 mM triethylamine acetate (pH 7.0) over 40 min at a flow rate of 3 ml/min. Compound **2** was eluted at 19 min, which was detected by measurement of absorption at 260 nm, and was then freeze-dried to give a white solid in 12% yield, which was calculated based on absorption at 260 nm compared with that of ATP. ^{1}H-NMR (400 MHz, D_2O, TSP): δ 8.57 (s, 1H), 8.27 (s, 1H), 6.16 (d, 1H, *J*=5.5 Hz), 4.54-4.61 (m, 1H), 4.41 (brs, 1H), 4.25 (brs, 2H), 2.63-2.74 (m, 2H), 2.32 (s, 1H), 1.90-1.95 (m, 2H), 1.57 (t, 2H, *J*=7.3 Hz), 1.50 ppm (t, 2H, *J*=6.9 Hz); HRMS (ESI$^-$): [M-H]$^-$ = 625.0727 calcd for $C_{17}H_{25}N_8O_{12}P_3$, found 625.0732.

Subsequently, to synthesize compound **3**, the reaction mixture of compound **2** described above without purification was added to a solution of tris[(1-benzyl-1*H*-1,2,3-triazol-4-yl)methyl]amine (13.2 mg, 50 µmol) and $CuSO_4 \cdot H_2O$ (3.12 mg, 25 µmol) in 1 ml water : DMF : *tert*-BuOH (3:6:1). After being stirred for 30 min, *N*-[2-[2-[2-(2-

azidoethoxy)ethoxy]ethoxy]ethyl]biotinamide (44.0mg, 100 µmol) and sodium ascorbate (49.5 mg, 250 µmol) were added to the solution followed by stirring for 30 min at room temperature. Compound **3** was purified by reverse-phase HPLC on ODS eluted at 22 min (17%), as described above. ^{1}H-NMR (400 MHz, D_2O, TSP): δ 8.27 (brs, 1H), 7.88 (brs, 1H), 6.21 (brs, 1H), 4.52-4.63 (m, 4H), 4.36-4.45 (m, 2H), 4.22-4.32 (m, 1H), 3.91-4.00 (m, 2H), 3.53-3.68 (m, 12H), 3.33-3.41 (m, 2H), 3.26-3.31 (m, 1H), 2.96 (dd, 1H, *J*=4.1, 11.8 Hz), 2.68-2.84 (m, 2H), 2.41 (brs, 1H), 2.24 (t, 2H, 6.9 Hz), 2.15 (s, 1H), 1.45-1.76 (m, 8H), 1.32-1.41 ppm (m, 2H); HRMS (ESI$^-$): $[M-H]^-$ = 1069.2881 calcd for $C_{35}H_{56}N_{14}O_{17}P_3S$, found 1069.2902.

Photoaffinity labeling

ABCD4(Y319C)-liposomes were incubated with or without AMP-PNP at 37°C for 30 min. The ATP probe (final conc. 0.2 mM) was then added and the mixtures incubated at 4°C for 30 min. After incubation, the ABCD4(Y319C) labeled by the ATP probe was photoactivated by UV irradiation at 360 nm for 30 sec. Samples were subjected to immunoblot analysis. His-ABCD4(Y319C) and the ATP probe were detected using an anti-His antibody and HRP-streptavidin, respectively.

Table S1 List of oligonucleotide primers

Primer name	5'- sequence-3'
Fw-ABCD4-N141K	GATGACATCGATAAGCCGGACCAGC
Fw-ABCD4-N141A	GATGACATCGATGCCCCGGACCAGC
Fw-ABCD4-N141D	GATGACATCGATGACCCGGACCAGC
Rv-ABCD4-N141	CCGCAGCACGTTGAGGGTGTAGTAC
Fw-ABCD4-Y319C	CTTTGTGTGCATCTGCCTCATCAGCTGC
Fw-ABCD4-Y319A	CTTTGTGTGCATCGCACTCATCAGCTG
Fw-ABCD4-Y319F	CTTTGTGTGCATCTTCCTCATCAGCTG
Rv-ABCD4-Y319	GCATTCTTGCTGACCAGGGTGCTAAG
Fw-ABCD4-K427A	GACCTCCTTGCTCCGGGTTCTGGG
Rv-ABCD4- K427A	CGGCCAGTGCCCGTGTTGCCTG
Fw-inv-LMBD1	CCTCCCAAAATGACTGCAGGCATGCAAGCTTCTTAGAC
Rv-inv-LMBD1	GGGGACATGGATCCGGCGGAGTAGACAGAAGGTTCG
Fw-infu-GST	CTCCGCCGGATCCATGTCCCCTATACTAGG
Rv-infu-GST	GCCTGCAGTCATTTTGGAGGATGGTCGCC

References

1. Okamoto, T., Kawaguchi, K., Watanabe, S., Agustina, R., Ikejima, T., Ikeda, K., Nakano, M., Morita, M., and Imanaka, T. (2018) Characterization of human ATP-binding cassette protein subfamily D reconstituted into proteoliposomes. *Biochemical and biophysical research communications* **496**, 1122-1127
2. Kawaguchi, K., Okamoto, T., Morita, M., and Imanaka, T. (2016) Translocation of the ABC transporter ABCD4 from the endoplasmic reticulum to lysosomes requires the escort protein LMBD1. *Scientific reports* **6**, 30183
3. Yokoyama, T., Hanawa, Y., Obita, T., and Mizuguchi, M. (2017) Stability and crystal structures of His88 mutant human transthyretins. *FEBS letters* **591**, 1862-1871
4. Kashiwayama, Y., Seki, M., Yasui, A., Murasaki, Y., Morita, M., Yamashita, Y., Sakaguchi, M., Tanaka, Y., and Imanaka, T. (2009) 70-kDa peroxisomal membrane protein related protein (P70R/ABCD4) localizes to endoplasmic reticulum not peroxisomes, and NH_2-terminal hydrophobic property determines the subcellular localization of ABC subfamily D proteins. *Experimental cell research* **315**, 190-205

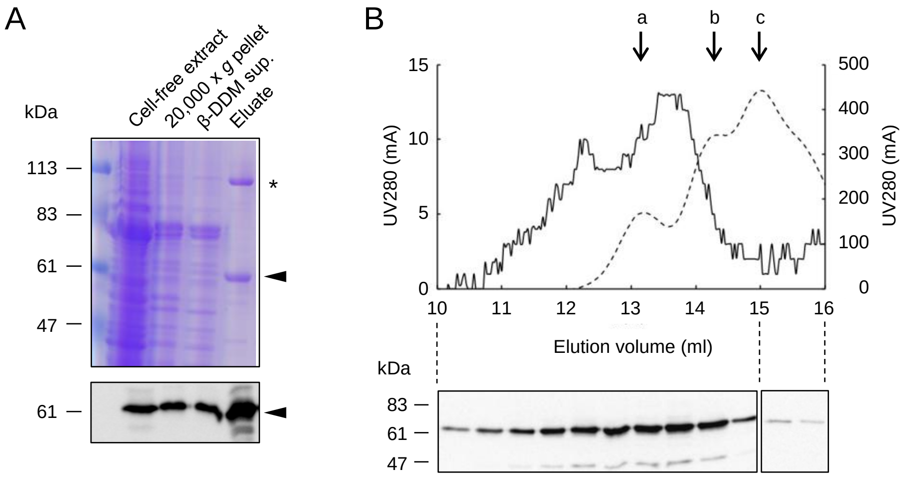

Supplementary Figure 1. Purification of His-ABCD4 expressed in *K. phaffii*.
(A) Cell lysate of *K. phaffii* expressing His-ABCD4 was subjected to centrifugation to obtain an organelle pellet. The membranes were solubilized by 0.5% β-DDM and the supernatant was incubated with cOmplete His-Tag Purification Resin. His-ABCD4 was subsequently eluted. Protein samples were subjected to SDS-PAGE followed by CBB staining (upper panel) and immunoblot (lower panel) using an anti-His antibody. The arrowhead and the asterisk indicate His-ABCD4 and a non-specific protein, respectively. (B) Gel filtration chromatograms of His-ABCD4 (solid) and molecular weight standards (dashed); a, Apoferritin (443 kDa); b, β-Amylase (200 kDa); c, Alcohol Dehydrogenase (150 kDa) (upper panel). Eluate was divided into 0.5 ml fractions. 30 μl of each fraction was subjected to SDS-PAGE followed by immunoblot using anti-His antibody (lower panel).

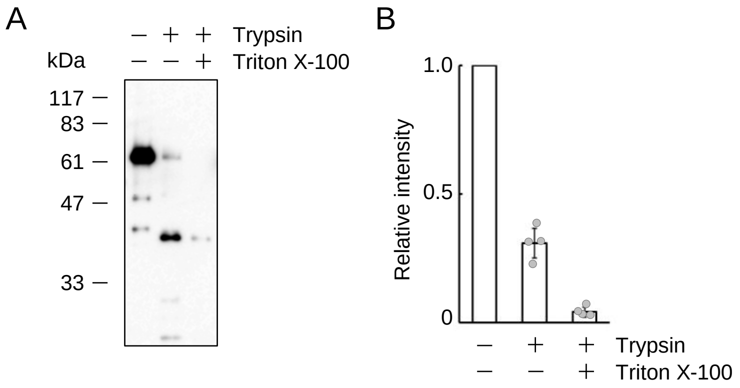

Supplementary Figure 2. Orientation of ABCD4 reconstituted into liposomes.
(A) Proteoliposomes containing ABCD4 were incubated with or without 50 ng of trypsin in the presence of 0.05% triton X-100 for 60 min on ice. ABCD4 was detected by immunoblot analysis using anti-ABCD4 antibody recognizing C-terminal 149 amino acids. (B) The amount of ABCD4 and its fragments was quantified using the image analysis software Image J. Signal intensity of ABCD4 without trypsin treatment has been normalized to 1. Error bars indicate the standard deviation (n=4).

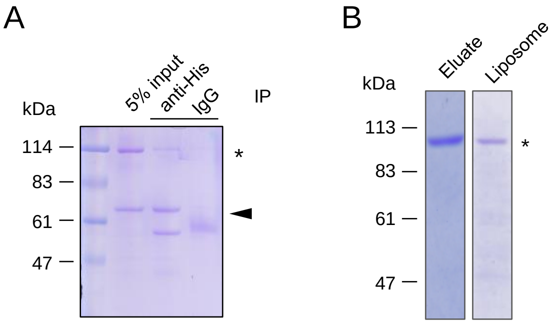

Supplementary Figure 3. Preparation of the negative control.
(A) The non-specific protein does not associate with ABCD4. The eluate fraction containing His-ABCD4 and non-specific protein was subjected to immunoprecipitation using an anti-His or control IgG. Precipitated proteins were analyzed by SDS-PAGE. The arrowhead and the asterisk indicate His-ABCD4 and a non-specific protein, respectively. (B) *K. phaffii* SMD1168, the host strain of heterologous ABCD4 expression, was used to prepare the purified fraction by the same procedure as the purification of His-ABCD4, and the non-specific protein in the eluate fraction was reconstituted in liposomes. Each fraction was subjected to SDS-PAGE and CBB staining. The asterisk indicates a non-specific protein.

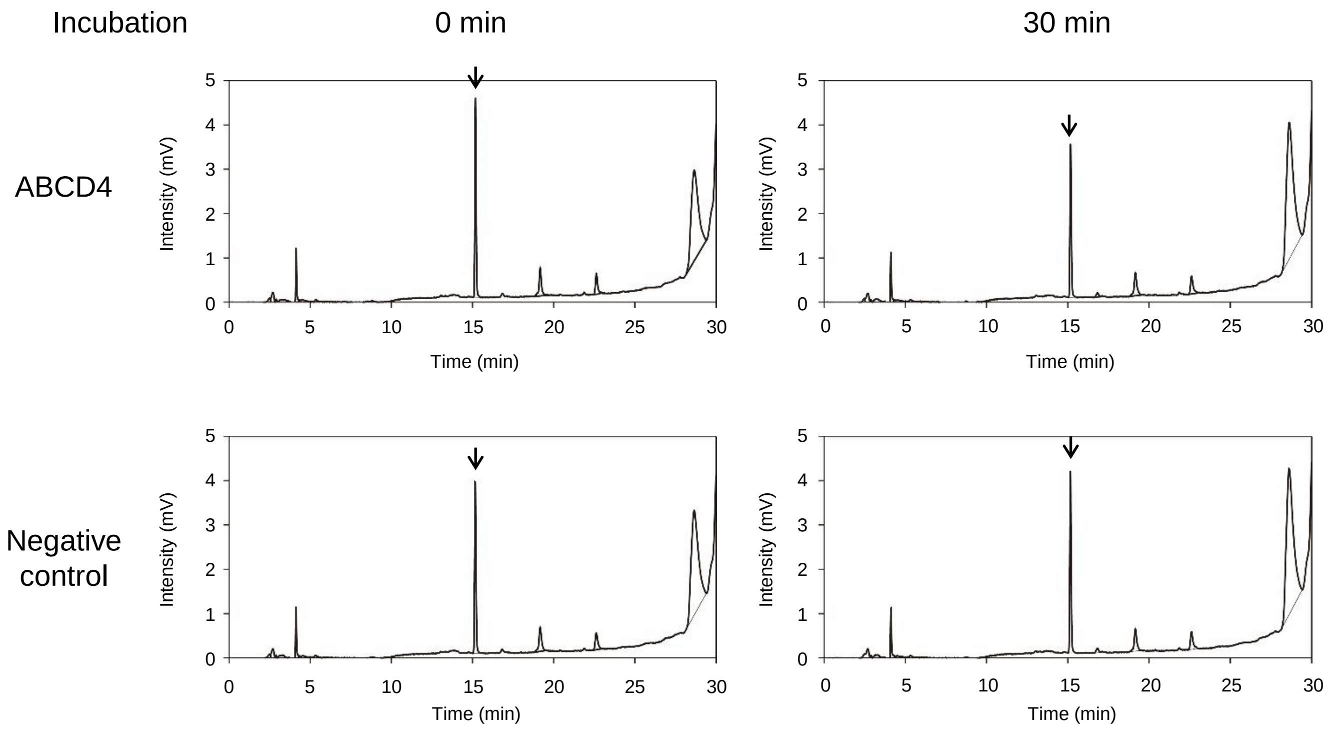

Supplementary Figure 4. HPLC analysis of cobalamin contained within liposomes. ABCD4-liposomes or negative control-liposomes containing cobalamin were incubated with ATP, and then each sample was collected using Sephadex G-50 column. Cobalamin was quantified by HPLC using reverse-phase column. The arrows indicate cobalamin. Cobalamin was released from ABCD4-liposomes but not negative control liposomes.

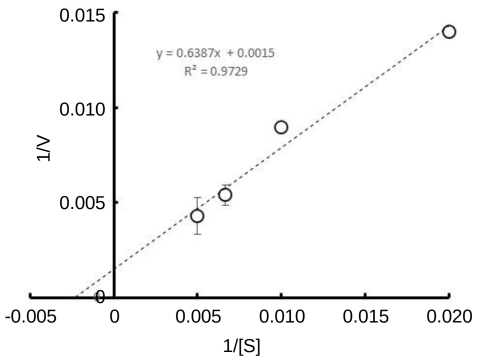

Supplementary Figure 5. Kinetic study of cobalamin transport by ABCD4 using Lineweaver-Burk plot.
ABCD4-liposomes loaded 50, 100, 150 or 200 μM of cobalamin were used to evaluate transport activities. The transport activity was plotted to 1/V and 1/S. Error bars indicate the standard deviation (n=3), when not shown, fall within the symbol.

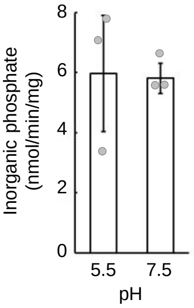

Supplementary Figure 6. Effect of the pH in the liposomes on the ATPase activity of ABCD4.

ABCD4-liposomes were prepared at pH 5.5 and pH7.5 and isolated by ultracentrifugation. Proteoliposomes at pH5.5 or pH 7.5 containing ABCD4 (5.80 µg or 5.16 µg, respectively) were incubated with ATP at 37°C for 30 min. Error bars indicate the standard deviation (n=3).

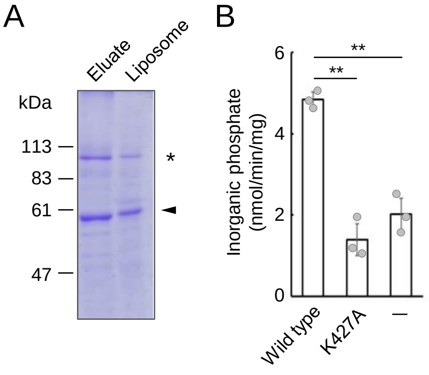

Supplementary Figure 7. ATPase activity of ABCD4(K427A).
(A) His-ABCD4(K427A) was prepared using the same procedure as the purification of wild type His-ABCD4. Purified ABCD4(K427A) and reconstituted ABCD4(K427A) were subjected to SDS-PAGE, and the gel was stained with CBB. The arrowhead and the asterisk indicate His-ABCD4(K427A) and a non-specific protein, respectively. (B) ATPase activity of reconstituted ABCD4(K427A) was measured. Proteoliposomes containing ABCD4 wild type or K427A (5.54 µg or 4.95 µg, respectively) were incubated with ATP at 37°C. Error bars indicate the standard deviation (n=3). Differences among the wild type, K427A and negative control were considered significant when $p<0.01$ based on Student's *t*-test (**$p<0.01$).

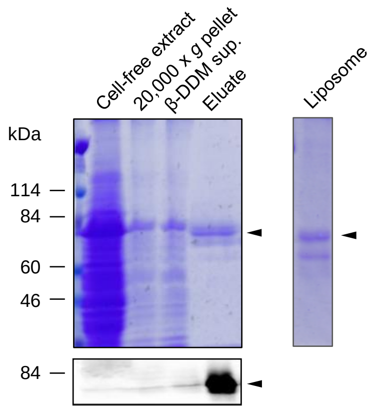

Supplementary Figure 8. Purification of LMBD1-GST expressed in *K. phaffii*.
Cell lysate of *K. phaffii* expressing LMBD1-GST was subjected to centrifugation to obtain an organelle pellet. The membranes were solubilized by 0.5% β-DDM and the supernatant was incubated with COSMOGEL GST-Accept agarose. LMBD1-GST was subsequently eluted. Protein samples were subjected to SDS-PAGE followed by CBB staining (upper panel) and immunoblot (lower panel) using an anti-GST antibody. Purified LMBD1-GST was reconstituted in liposomes (right panel). The arrowhead indicates LMBD1-GST.

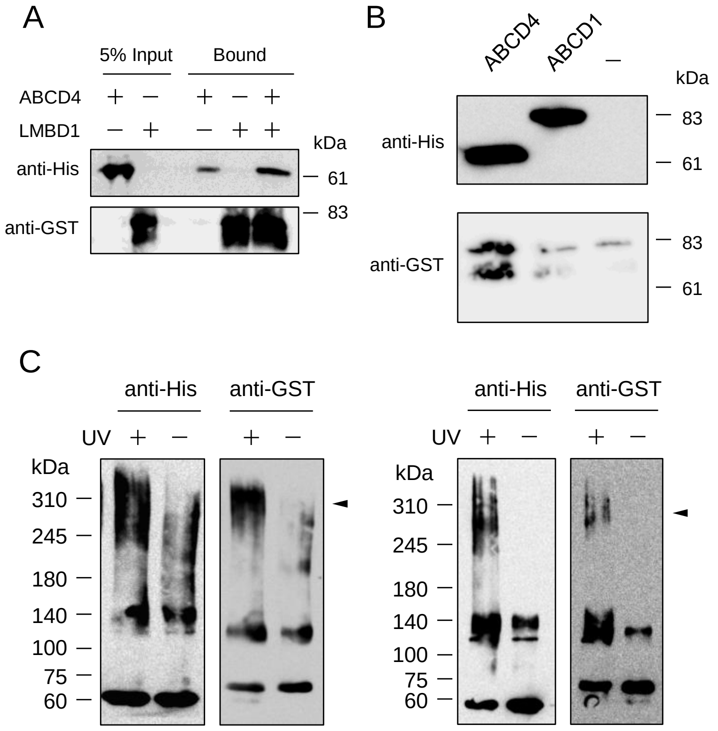

Supplementary Figure 9. Interaction analysis between purified ABCD4 and LMBD1. (A) The eluates of His-ABCD4 and LMBD1-GST were mixed and subjected to pull-down assay using COSMOGEL GST-Accept agarose. Co-precipitated proteins were analyzed by SDS-PAGE followed by immunoblotting using an anti-His or an anti-GST antibody. (B) LMBD1-GST was mixed with His-ABCD4 or His-ABCD1 and subjected to pull-down assay using His-tag affinity resin. Co-precipitated proteins were analyzed by SDS-PAGE followed by immunoblotting using an anti-His or an anti-GST antibody. (C) Binding stoichiometry between ABCD4 and LMBD1. Purified His-ABCD4 and LMBD1-GST were incubated and then subjected to photocrosslinking using SDA and MBS. His-ABCD4 and LMBD1-GST were detected by immunoblot analysis with an anti-His antibody and an anti-GST antibody, respectively (left panel). Binding stoichiometry of ABCD4/LMBD1 complex in liposomes was also analyzed using the same procedure (right panel). The arrowhead indicates ABCD4/LMBD1 complex.

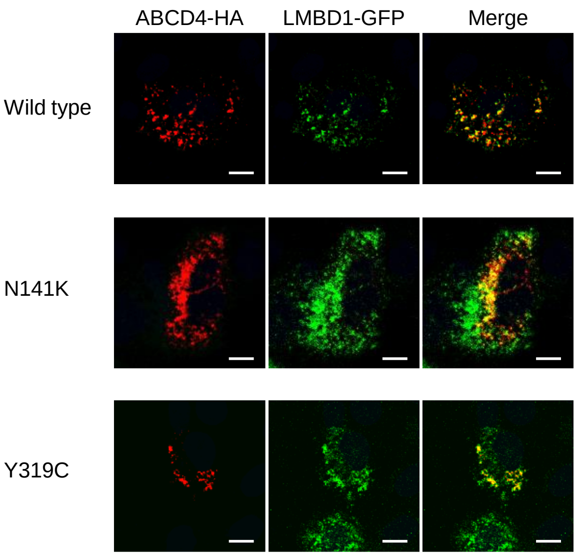

Supplementary Figure 10. Subcellular localization of mutant ABCD4-HA co-expressed with LMBD1-GFP in CHO cells.
HA-tagged ABCD4s were transiently expressed in CHO cells stably expressing LMBD1-GFP. The distribution of ABCD4 that was detected by immunofluorescence staining with an anti-HA antibody was compared with the LMBD1-GFP which localized on the lysosomal membranes. Bar, 10 μm.

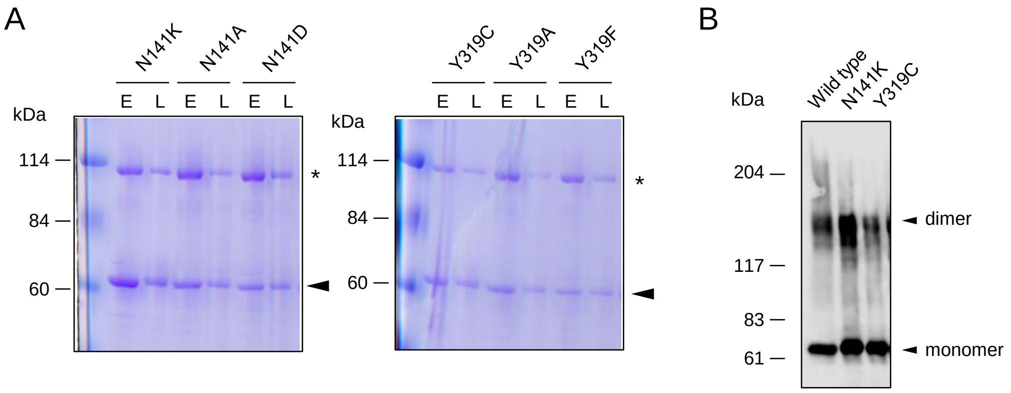

Supplementary Figure 11. Purification of mutant ABCD4s.

(A) His-tagged mutant ABCD4s were prepared using the same procedure as in the purification of His-ABCD4. Purified mutant ABCD4s and reconstituted mutant ABCD4s were subjected to SDS-PAGE, and the gels were stained with CBB. The arrowhead and the asterisk indicate mutant ABCD4s and a non-specific protein, respectively. E: Eluate, L: liposomes (B) Photocrosslinking of His-ABCD4s was performed with SDA and UV irradiation. His-ABCD4s was detected by immunoblot analysis with an anti-His antibody.

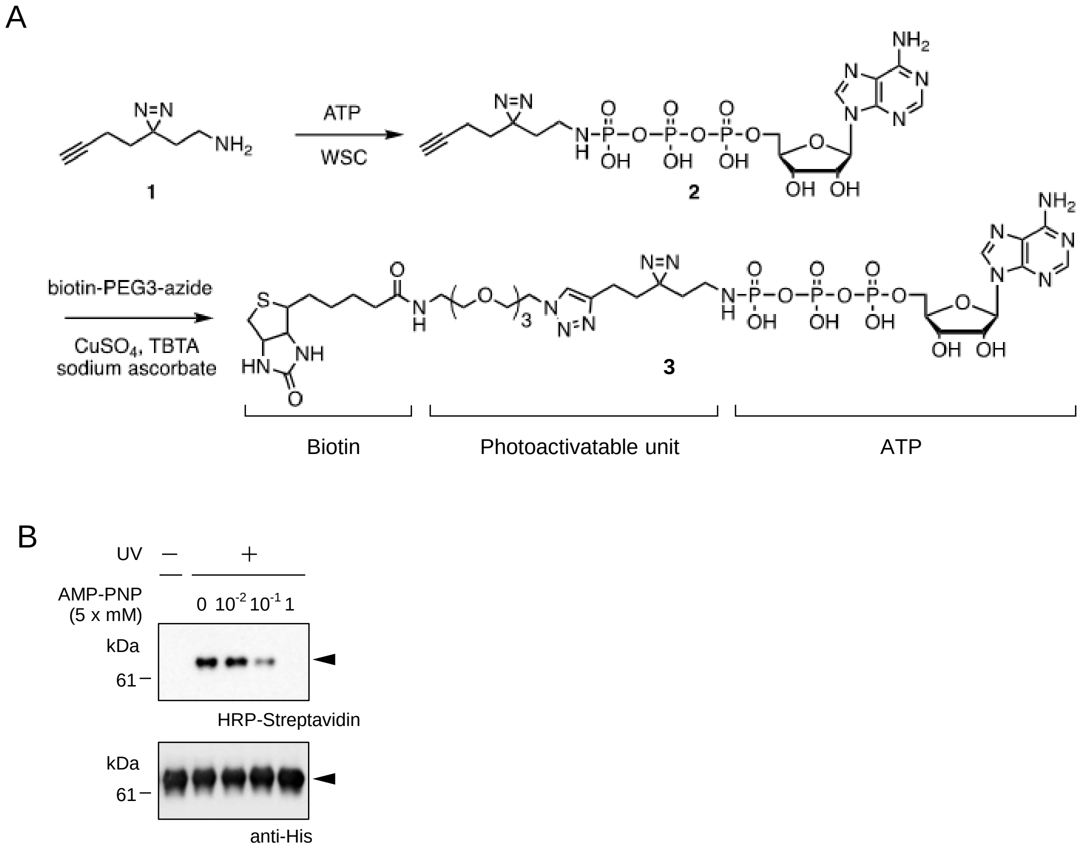

Supplementary Figure 12. Photoaffinity labeling of ABCD4(Y319A) with the ATP probe. (A) A schematic procedure for the synthesis of ATP probe. (B) Photoaffinity labeling of ABCD4(Y319A) with the ATP probe under irradiation with 360 nm light at 0°C in the absence or presence of AMP-PNP. The photoproducts were subjected to SDS-PAGE followed by immunoblotting using an HRP-streptavidin (upper panel) or anti-His antibody (lower panel). The arrowheads indicate His-ABCD4(Y319C).

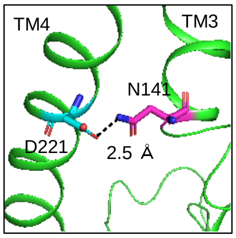

Supplementary Figure 13. The local structure around Asn^{141} and Asp^{221}.
This model is drawn based on the atomic coordinates of ABCD4 deposited in the Protein Data Bank (PDB code: 6JBJ). Asn^{141} and Asp^{221} are colored in magenta and light blue, respectively. The distance between Asn^{141} and Asp^{221} was evaluated using the molecular modeling software PyMOL.
